# ﻿Additions to the genus *Kirschsteiniothelia* (Dothideomycetes); Three novel species and a new host record, based on morphology and phylogeny

**DOI:** 10.3897/mycokeys.110.133450

**Published:** 2024-10-28

**Authors:** Xia Tang, Rajesh Jeewon, Ruvishika S. Jayawardena, Deecksha Gomdola, Yong-Zhong Lu, Rong-Ju Xu, Abdulwahed Fahad Alrefaei, Fatimah Alotibi, Kevin D. Hyde, Ji-Chuan Kang

**Affiliations:** 1 Engineering and Research Center for Southwest Biopharmaceutical Resource of National Education Ministry of China, Guizhou University, Guiyang, 550025, Guizhou Province, China; 2 Center of Excellence in Fungal Research, Mae Fah Luang University, Chiang Rai, 57100, Thailand; 3 School of Science, Mae Fah Luang University, Chiang Rai, 57100, Thailand; 4 Department of Health Sciences, Faculty of Medicine and Health Sciences, University of Mauritius, Reduit, Mauritius; 5 School of Food and Pharmaceutical Engineering, Guizhou Institute of Technology, Guiyang, Guizhou Province 550003, China; 6 Department of Zoology, College of Science, King Saud University, P.O. Box 2455, Riyadh 11451, Saudi Arabia; 7 Department of Botany and Microbiology, College of Science, King Saud University, P.O. Box 22452, 11495 Riyadh, Saudi Arabia

**Keywords:** Checklist, diversity, Dothideomycetes, Kirschsteiniotheliales, one new host record, taxonomy, three new taxa

## Abstract

During a survey of microfungi associated with forest plants, four specimens related to *Kirschsteiniothelia* were collected from decaying wood in Guizhou, Hainan and Yunnan Provinces, China. *Kirschsteiniothelia* species have sexual and asexual forms. They are commonly found as saprophytes on decaying wood and have been reported as disease-causing pathogens in humans as well. In this study, we introduce three novel *Kirschsteiniothelia* species (*K.bulbosapicalis*, *K.dendryphioides* and *K.longirostrata*) and describe a new host record for *K.atra*, based on morphology and multi-gene phylogenetic analyses of a concatenated ITS, LSU and SSU rDNA sequence data. These taxa produced a dendryphiopsis- or sporidesmium-like asexual morph and detailed descriptions and micromorphological illustrations are provided. Furthermore, we provide a checklist for the accepted *Kirschsteiniothelia* species, including detailed host information, habitat preferences, molecular data, existing morphological type, country of origin and corresponding references.

## ﻿Introduction

*Kirschsteiniothelia* was introduced by Hawksworth (1985) and typified by *K.aethiops*, based on morphological observation, linking it to its asexual genus, *Dendryphiopsis* S. Hughes. Later, the type species was reclassified with its asexual morph, *K.atra* (Hyde et al 2011; Wijayawardene et al. 2014). The connection between the sexual morphs of *Kirschsteiniotheliaatra* (characterised by cylindrical-clavate, bitunicate, 8-spored or rarely 4-spored asci and ellipsoidal verruculose or smooth ascospores with 1–2 septa, lacking a distinct gelatinous sheath) and the asexual morphs (characterised by macronematous, mononematous and branched conidiophores, monotretic, terminal or intercalary, cylindrical, doliiform conidiogenous cells and acrogenous, solitary, cylindrical, oblong, septate conidia with obtuse ends) were previously established by Hughes (1978). This connection was confirmed from cultures obtained from fragments of the ascomata, based on morphological examination. Schoch et al. (2006) further confirmed the connection between the sexual and asexual morphs of *Kirschsteiniothelia*, based on both morphology and phylogenetic analysis of SSU, LSU, *tef1-α* and *rpb2*. Boonmee et al. (2012) established a novel family, Kirschsteiniotheliaceae, based on the connection between the sexual and asexual morph of *Kirschsteiniothelia* and multiple gene (LSU, SSU and ITS) phylogeny. Wijayawardene et al. (2014) suggested the use of *Kirschsteiniothelia* as the updated genus to accommodate *Dendryphiopsis* species. As a result, *Dendryphiopsisatra* was re-assigned to *Kirschsteiniothelia* and synonymised with *K.atra*, based on morphological and phylogenetic analyses. In the meantime, Wijayawardene et al. (2014) suggested using *K.atra* to replace *K.aethiops* as the type species of *Kirschsteiniothelia*. This recommendation was supported by later studies (Rossman et al. 2015; Su et al. 2016; Bao et al. 2018; Yang et al. 2023; Jin et al. 2024; Sruthi et al. 2024; Tian et al. 2024). Sruthi et al. (2024) legitimately placed five species from *Dendryphiopsis* under *Kirschsteiniothelia*, namely, *K.arbuscula*, *K.binsarensis*, *K.biseptata*, *K.fascicularis* and *K.goaensis*. *Kirschsteiniothelia* usually exhibits both sexual and asexual morphs (Hawksworth 1985; Boonmee et al. 2012; Hyde et al. 2013; Sun et al. 2021; Xu et al. 2023). The sexual morph of *Kirschsteiniothelia* is characterised by superficial, erumpent, papillate, brown or black and hemi-spherical or subglobose ascomata and cylindrical or clavate, bitunicate, pedicellate asci that are usually 8-spored comprising an ocular apical chamber. The ascospores are ellipsoidal, usually asymmetrical, verruculose or smooth and olivaceous to dark brown, comprising 1–2 septa, with a mucilaginous sheath being occasionally present (Chen et al. 2006; Hyde et al. 2018; Meng et al. 2024). Furthermore, ascospores occasionally display longitudinal or sinuate furrows that are visible from the face view (Hawksworth 1985; Boonmee et al. 2012; Hyde et al. 2013; Mehrabi et al. 2017; Yang et al. 2023).

The asexual morph is further categorised into two types, namely the dendryphiopsis- and sporidesmium-like morphs. The dendryphiopsis-like morph was described by Hughes (1978), who found that the ascomatal fragments of *Kirschsteiniotheliaaethiops* (≡ *Amphisphaeriaincrustans*) exhibited agar sporulation and morphological traits similar to those of *Dendryphiopsisatra*. This was later supported by Hawksworth (1985). Subsequently, Boonmee et al. (2012) supported the connection between the sexual morph of *Kirschsteiniothelia* and the asexual dendryphiopsis-like morph, based on morphological and phylogenetic analyses. The dendryphiopsis-like morph is characterised by macronematous, septate, cylindrical conidiophores that are irregularly or subscorpioidly branched at the apex. Their conidiogenous cells are mono- to polytretic, integrated, terminal or lateral, doliiform or lageniform. Moreover, the conidia are holoblastic, acrogenous, obclavate, rostrate, obovoid to broadly obovoid, solitary or branched in acropetal chains, exhibiting rounded ends. Taxa exhibiting the dendryphiopsis-like characteristics are *K.atra*, *K.arbuscula*, *K.binsarensis*, *K.biseptata*, *K.dendryphioides*, *K.ebriosa*, *K.emarceis*, *K.fascicularis*, *K.goaensis*, *K.inthanonensis*, *K.lignicola*, *K.longisporum*, *K.nabanheensis*, *K.ramus*, *K.recessa*, *K.saprophytica*, *K.septemseptatum*, *K.shimlaensis*, *K.vinigena* and *K.zizyphifolii* (Hughes 1978; Boonmee et al. 2012; de Farias et al. 2024; Tian et al. 2024; this study).

The sporidesmium-like asexual morph was described by Su et al. (2016), based on morphological and phylogenetic evidence. Despite having different morphological characteristics from other *Kirschsteiniothelia* species, the sporidesmium-like morphs fits into the generic concept of *Kirschsteiniothelia* as they display similar morphologies including unbranched, slender conidiophores that are straight or slightly curved, multi-septate and brown to pale brown, usually truncate at the base and rounded at the apex, producing small conidia. The sporidesmium-like morph is depicted by macronematous, mononematous, unbranched, multi-septate, cylindrical conidiophores, holoblastic, integrated, terminal, determinate, percurrent, cylindrical and caliciform conidiogenous cells and acrogenous, multi-septate, obclavate to obspathulate, rostrate and fusiform conidia that are swollen at the tips or middle of the beak, with or without a conspicuous, gelatinous, hyaline sheath around the tip or middle of the beak. The presence of the sporidesmium-like asexual morph of *Kirschsteiniothelia* was further supported by subsequent research (Sun et al. 2021; Jayawardena et al. 2022; Xu et al. 2023; Yang et al. 2023). Species exhibiting the sporidesmium-like features are *K.acutispora*, *K.agumbensis*, *K.aquatica*, *K.bulbosapicalis*, *K.cangshanensis*, *K.crustacea*, *K.dujuanhuensis*, *K.dushanensis*, *K.extensum*, *K.fluminicola*, *K.guangdongensis*, *K.longirostrata*, *K.pini*, *K.puerensis*, *K.rostrata*, *K.sichuanensis*, *K.spatiosum*, *K.submersa*, *K.tectonae*, *K.thailandica* and *K.xishuangbannaensis* (Su et al. 2016; Jayawardena et al. 2022; Xu et al. 2023; Yang et al. 2023; Jin et al. 2024; Sruthi et al. 2024; this study).

Although *Kirschsteiniothelia* comprises numerous species, there are likely to be more undescribed species in this genus as predicted by Bhunjun et al. (2022). Most species have been reported as saprobes inhabiting terrestrial and freshwater environments in tropical and subtropical regions. However, *K.ebriosa* and *K.vinigena* have been identified from cork taint of sparkling wine (Bao et al. 2018; Rodríguez-Andrade et al. 2020; Sun et al. 2021; Jayawardena et al. 2022). Moreover, a report indicates the presence of an unidentified *Kirschsteiniothelia* species that is pathogenic to humans, causing infection superimposed on pre-existing non-infectious bursitis of the ankle. This identification was based on the examination of the strain’s cultural colony and ITS gene fragment (Nishi et al. 2018). To date, there are 59 species of *Kirschsteiniothelia*, amongst which 18 have been reported only in their sexual morph, 32 reported in their asexual morph and six species documented in both morphs (Boonmee et al. 2012; Su et al. 2016; Sun et al. 2021; Xu et al. 2023; Zhang et al. 2023; de Farias et al. 2024; Sruthi et al. 2024; this study). Amongst the two asexual morphs that have been described so far, only the dendryphiopsis-like morph is linked to the sexual morph, while the sporidesmium-like state has not been associated with the sexual morph (Hawksworth 1985; Wang et al. 2004; Mulenko et al. 2008; Boonmee et al. 2012; Su et al. 2016; Sun et al. 2021; Xu et al.2023; de Farias et al. 2024).

In this study, we aimed to isolate microfungi from unidentified decaying wood collected in Hainan and Yunnan Provinces, China, as well as from *Edgeworthiachrysantha*, collected in Guizhou Province, China. This study has the following objectives: 1) to describe novel species associated with decaying wood through comprehensive morphological examinations and phylogenetic analyses of ITS, LSU and SSU rDNA sequence data; 2) to provide a checklist that includes host information, habitat preferences, availability of molecular data, morphological characteristics and country of origin.

## ﻿Materials and methods

### ﻿Sample collection, isolation and morphological studies

Decaying wood materials of *Edgeworthiachrysantha* and unidentified plants were collected from Zunyi City in Guizhou Province, Jianfengling National Forest Park, situated at the confluence of Ledong Li Autonomous County and Dongfang City in Hainan Province and Lushui City in Yunnan Province, China. These specimens were initially stored in Ziploc bags and observed using a stereomicroscope (Motic SMZ-171). The collection, observation and isolation were conducted following the methods outlined in Senanayake et al. (2020) and Tang et al. (2022). The observed features were measured using Tarosoft (R) Image Frame Work (version IFW 0.97) and photoplates were constructed using Adobe Photoshop 2019 (Adobe Systems, USA).

Specimens were deposited at the herbaria of the Kunming Institute of Botany, Chinese Academy of Sciences (HKAS), located in Kunming, China and the Guizhou Academy of Agriculture Sciences (GZAAS), situated in Guiyang, China. In addition, ex-type living cultures were preserved at the Kunming Institute of Botany Culture Collection (KUMCC) and the Guizhou Culture Collection (GZCC). Faces of Fungi and Fungal name numbers were obtained following the guidelines in Jayasiri et al. (2015), Wang et al. (2023) and Fungal names (2024). Species identification and establishment were determined following the guidelines outlined by Jeewon and Hyde (2016), Maharachchikumbura et al. (2021) and Pem et al. (2021).

### ﻿DNA extraction, PCR amplification and sequencing

Freshly scraped mycelia from the pure cultures obtained by single spore isolation were transferred to 1.5 ml microcentrifuge tubes and stored in the refrigerator at -20 °C. Genomic DNA extraction was carried out using DNA extraction kits provided by Sangon Biotech (Shanghai) Co. Ltd., China. Polymerase Chain Reaction (PCR) was employed for DNA template amplification, using the following primer pairs: ITS5/ITS4 for ITS, NS1/NS4 for SSU (White et al. 1990) and LR0R/LR5 for LSU (Vilgalys and Hester 1990; Cubeta et al. 1991). Further details regarding DNA extraction, PCR amplification, sequencing and phylogenetic analyses are given in Tang et al. (2022, 2023).

In PCR amplification, the total volume of the PCR mixture was 50 μl, comprising the DNA template (2 μl), forward primer (2 μl), reverse primer (2 μl), 2 × Taq PCR Master Mix (25 μl) and 19 μl of double-distilled water. The PCR profiles consisted of 35 cycles, with annealing temperatures set at 52 °C for 1 minute and extension for 90 seconds at 72 °C for ITS, LSU and SSU loci. PCR products were verified on 1% agarose gel prior to submission to Sangon Biotech (Shanghai) Co., Ltd., China, for sequencing.

### ﻿Phylogenetic analyses

Sequences obtained were subjected to a BLAST search in the NCBI database (https://blast.ncbi.nlm.nih.gov/Blast.cgi). Forward and reverse sequences were assembled using the Contig Express version 3.0.0 application. The ITS, LSU and SSU sequence data of *Kirschsteiniothelia* species were retrieved and downloaded from GenBank (Table 1). Individual sequences were aligned using MAFFT version 7 (https://mafft.cbrc.jp/alignment/server/index.html) with the “auto” option (Katoh et al. 2017). The aligned sequences were trimmed using trimAl version 1.2 with the ‘-gt 0.6’ command (Capella-Gutiérrez et al. 2009) and multiple genes were assembled using SequenceMatrix (Vaidya et al. 2011).

**Table 1.** Taxa used in this study and their respective GenBank accession numbers.

Taxon	Strain number	ITS	LSU	SSU
*Kirschsteiniotheliaacutispora*	MFLU 21-0127^T^	OP120780	ON980758	ON980754
*K.atra*	CBS 109.53	–	AY016361	AY016344
*K.atra*	MFLUCC 15-0424	KU500571	KU500578	KU500585
*K.atra*	MFLUCC 16-1104	MH182583	MH182589	MH182615
*K.atra*	S-783	MH182586	MH182595	MH182617
*K.atra*	GZCC 23-0731	PQ248940	PQ248936	PQ248932
*K.atra*	DEN^T^	MG602687	–	–
*K.aquatica*	MFLUCC 16-1685^T^	MH182587	MH182594	MH182618
*K.arasbaranica*	IRAN 2509C	KX621986	KX621987	KX621988
*K.arasbaranica*	IRAN 2508C^T^	KX621983	KX621984	KX621985
*K.agumbensis*	NFCCI 5714^T^	PP029048	–	PP029049
*K.bulbosapicalis*	GZCC 23-0732^T^	PQ248937	PQ248933	PQ248929
*K.cangshanensis*	GZCC19-0515	–	MW133829	MW134609
*K.cangshanensis*	MFLUCC 16-1350^T^	MH182584	MH182592	–
*K.chiangmaiensis*	MFLU 23-0358^T^	OR575473	OR575474	OR575475
*K.crustacea*	MFLU 21-0129^T^	MW851849	MW851854	
*K.dendryphioides*	KUNCC 10431^T^	OP626354	PQ248935	PQ248931
*K.dendryphioides*	KUNCC 10499	PQ248938	–	–
*K.dujuanhuensis*	KUNCC 22-12671	OQ874971	OQ732682	
*K.dushanensis*	GZCC 19-0415^T^	OP377845	MW133830	MW134610
*K.ebriosa*	CBS H–23379^T^	–	LT985885	–
*K.emarceis*	MFLU 10-0037^T^	NR_138375	NG_059454	–
*K.esperanzae*	T. Raymundo 6581^T^	OQ877253	OQ880482	–
*K.extensum*	MFLU 21-0130^T^	MW851850	MW851855	–
*K.fluminicola*	MFLUCC 16-1263^T^	MH182582	MH182588	–
*K.guangdongensis*	ZHKUCC 22-0233^T^	OR164946	OR164974	–
*K.inthanonensis*	MFLUCC 23-0277^T^	OR762773	OR762781	OR764784
*K.laojunensis*	KUN-L 88727^T^	PP081651	PP081658	–
*K.lignicola*	MFLUCC 10-0036^T^	HQ441567	HQ441568	HQ441569
*K.longirostrata*	GZCC 23-0733^T^	PQ248939	PQ248934	PQ248930
*K.longisporum*	UESTCC 24.0190^T^	PQ038266	PQ038273	PQ046108
*K.nabanheensis*	HJAUP C2006	OQ023274	OQ023275	OQ023037
*K.nabanheensis*	HJAUP C2004^T^	OQ023197	OQ023273	OQ023038
*K.phoenicis*	MFLU 18-0153	NR_158532	NG_064508	–
*K.phoenicis*	MFLUCC 18-0216^T^	MG859978	MG860484	MG859979
*K.pini*	UESTCC 24.0131^T^	PP835321	PP835315	PP835318
*K.puerensis*	ZHKUCC 21-0271^T^	OP450977	OP451017	OP451020
*K.puerensis*	ZHKUCC 22-0272	OP450978	OP451018	OP451021
*K.ramus*	GZCC 23-0596^T^	OR098711	OR091333	–
*K.rostrata*	MFLUCC15-0619	KY697280	KY697276	NG_063633
*K.rostrata*	MFLU 15-1154	NR_156318	NG_059790	KY697278
*K.rostrata*	MFLUCC 16-1124	–	MH182590	–
*K.saprophytica*	MFLUCC 23-0276	OR762775	OR762782	–
*K.saprophytica*	MFLUCC 23-0275^T^	OR762774	OR762783	–
*K.septemseptatum*	MFLU 21-0126^T^	OP120779	ON980757	ON980752
*K.sichuanensis*	UESTCC 24.0127^T^	PP785368	PP784322	–
*Kirschsteiniothelia* sp.	KUNCC 23-13755	OR589301	–	–
*Kirschsteiniothelia* sp.	KUNCC 23-14559	OR589302	–	–
*Kirschsteiniothelia* sp.	KUNCC 23-13756	OR589303	–	–
*Kirschsteiniothelia* sp.	E38	MN912317	MN912273	–
*Kirschsteiniothelia* sp.	CSN602	MT813880	–	–
*Kirschsteiniothelia* sp.	CSN604	MT813881	–	–
*Kirschsteiniothelia* sp.	UTHSCSA D122 44	–	ON191450	–
*Kirschsteiniothelia* sp.	UTHSCSA D122 45	–	ON191449	–
*Kirschsteiniothelia* sp.	7020611638	–	MZ380317	–
*K.spatiosum*	MFLU 21-0128^T^	OP077294	–	ON980753
*K.submersa*	S-601	MH182585	MH182593	–
*K.submersa*	S-481	–	MH182591	MH182616
*K.submersa*	MFLUCC 15-0427^T^	KU500570	KU500577	KU500584
*K.tectonae*	MFLUCC 12-0050^T^	KU144916	KU764707	–
*K.tectonae*	MFLUCC 13-0470	KU144924	–	–
*K.thailandica*	MFLUCC 20-0116^T^	MT985633	MT984443	MT984280
*K.thujina*	JF13210	KM982716	KM982718	KM982717
*K.vinigena*	CBS H-23378^T^	–	NG_075229	–
*K.xishuangbannaensis*	ZHKUCC 22-0221	OP289563	OP303182	OP289565
*K.xishuangbannaensis*	ZHKUCC 22-0220^T^	OP289566	OP303181	OP289564
*K.zizyphifolii*	MFLUCC 23-0270^T^	OR762768	OR762776	OR764779
*Strigulaguangxiensis*	HMAS-L0138040	NR146255	MK206256	–
*S.nemathora*	MPN 72	–	JN887405	JN887389

Notes: Ex-type strains are indicated by “T” in superscript and newly-generated sequences are in red. Abbreviations: **CBS**: Westerdijk Fungal Biodiversity Institute, Utrecht, Netherlands; **CSN**: collection of Chris Spies at ARC-Nietvoorbij, Stellenbosch, South Africa; **GZCC**: Guizhou Culture Collection, Guizhou, China; **HJAUP**: Herbarium of Jiangxi Agricultural University, Plant Pathology; **HMAS-L**: Fungarium of the Institute of Microbiology, Chinese Academy of Sciences; **IRAN**: Iranian Fungal Culture Collection, Iranian Research Institute of Plant Protection, Tehran, Iran; **JF**: Jacques Fournier; **KUNCC**: Kunming Institute of Botany Culture Collection; **KUN-L**: Lichen Herbarium of Kunming Institute of Botany, Chinese Academy of Science, Yunnan, China; **MFLUCC**: Mae Fah Luang University Culture Collection, Chiang Rai, Thailand; **MFLU**: Mae Fah Luang University Herbarium Collection; **NFCCI**: National Fungal Culture Collection of India NFCCI-A National Facility; **ZHKUCC**: Zhongkai University of Agriculture and Engineering Culture Collection, Guangzhou, China. *K.: Kirschsteiniothelia*; *S.: Strigula*; “–”: Data not available.

The phylogenetic analyses of the concatenated ITS, LSU and SSU sequences were conducted using Maximum Likelihood (ML) and Bayesian Inference (BI). Maximum Likelihood analysis was conducted using the IQ tree web server (http://iqtree.cibiv.univie.ac.at) and BI was carried out in the CIPRES web portal (Miller et al. 2010). The BI was performed using the tool “MrBayes on XSEDE” (Huelsenbeck and Ronquist 2001; Swofford 2002; Stamatakis et al. 2008; Ronquist et al. 2012). Prior to conducting BI, the model of evolution for each gene region was estimated using MrModelTest version 2 (Tang et al. 2023). The aligned Fasta file was converted into a Nexus format for subsequent Bayesian analysis using AliView version 1.27 (Daniel et al. 2010). Phylograms were visualised using FigTree version 1.4.0 and edited in the Adobe Photoshop 2019 programme (Adobe Systems, USA) and Adobe Illustrator version 51.1052.0.0 (Adobe Inc., San Jose, California, USA).

## ﻿Results

### ﻿Phylogenetic analyses

**Figure 1.**
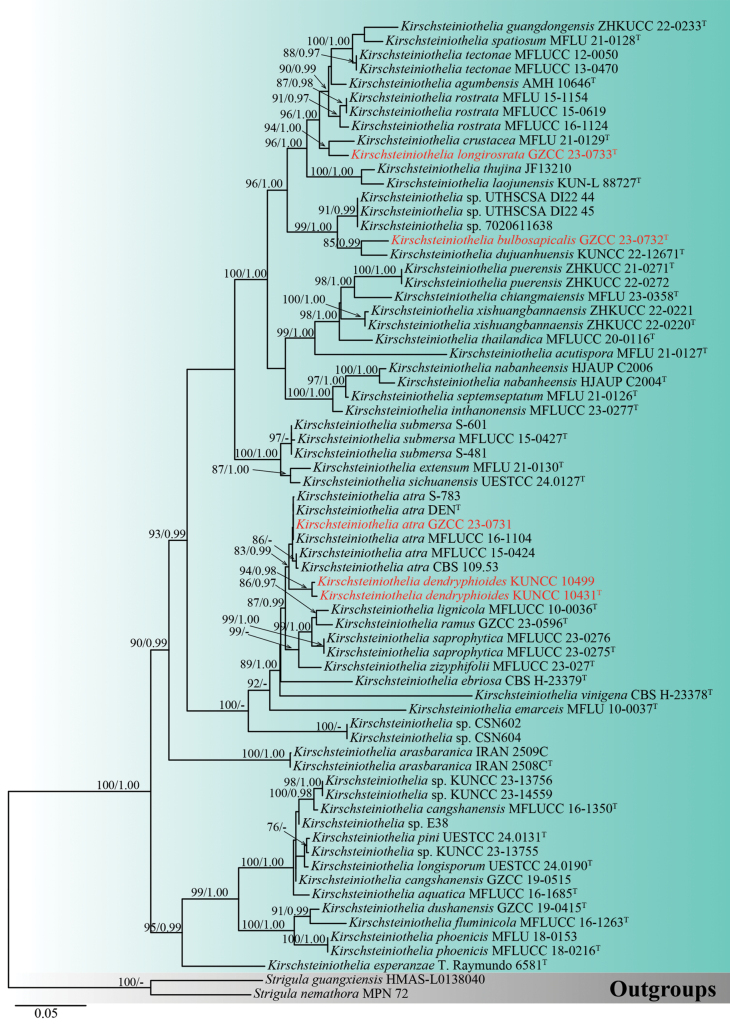
Phylogram of *Kirschsteiniothelia* taxa, based on the RAxML analysis of the combined ITS, LSU and SSU rDNA sequence dataset. Bootstrap support values for Maximum Likelihood (ML) equal to or greater than 75% and Bayesian posterior probabilities (PP) equal to or greater than 0.95 are shown above the nodes. The tree is rooted with *Strigulaguangxiensis* (HMAS-L0138040) and *S.nemathora* (MPN 72). Newly-generated strains are denoted in red and type strains are indicated with a superscript “T”.

According to the analysis of the concatenated ITS, LSU and SSU rDNA sequence data, all isolates collected in this study cluster within *Kirschsteiniothelia*. The dataset with 67 strains of *Kirschsteiniothelia*, including gaps, comprises 2290 characters (ITS: 1–506 base pairs (bp), LSU: 507–1330 bp and SSU: 1331–2283 bp). The highest-scoring RAxML tree is presented in Fig. 1, with a final ML optimisation likelihood value of -16683.670 (ln). The best-fit model for the BI analysis was GTR+I+G for ITS, LSU and SSU. Bayesian posterior probabilities (PP) from MCMC were analysed, achieving a final average standard deviation of split frequencies of 0.009914.

### ﻿Taxonomy

#### ﻿ Kirschsteiniothelia atra

Taxon classificationFungiPleosporalesKirschsteiniotheliaceae

(Corda) D. Hawksw., Fungal Diversity 69: 37 (2014)

A2C30658-7CE2-56C4-A2C1-734E7B9B1346

Fungal Names number: FN 104401

Facesoffungi number: FoF01738

Fig. 2

≡ Amphisphaeriaaethiops Sacc., Syll. fung. (Abellini) 1: 722 (1882)
= Dendryphiopsisatra (Corda) S. Hughes, Can. J. Bot. 31: 655 (1953)
≡ Dendryphionatrum Corda, Icon. fung. (Prague) 4: 33 (1840)
≡ Kirschsteiniotheliaaethiops (Sacc.) D. Hawksw., J. Linn. Soc., Bot. 91(1–2): 185 (1985)

##### Description.

***Saprobic*** on decaying wood of *Edgeworthiachrysantha*. ***Sexual morph***: see Hawksworth (1985). ***Asexual morph*: *Colonies*** on the natural substrate superficial, effuse, gregarious, dark brown to black, glistening. ***Mycelium*** immersed, composed of branched, septate, thin-walled, smooth, brown hyphae. ***Conidiophores*** 253–396 × 8–15.5 µm (x̄= 334.6 × 11.7 µm, n = 20), macronematous, mononematous, erect, straight or flexuous, cylindrical, septate, smooth, brown to dark brown, becoming paler towards the apex and comprising numerous short branches. ***Conidiogenous cells*** 14.5–29 × 5–10 µm (x̄= 20.6 × 6.8 µm, n = 30), tretic, integrated, sometimes percurrent, terminal, doliiform or lageniform, subhyaline to pale brown, with new cells developing from the apical or subapical part of the subtending cells. ***Conidia*** 32–56.5 × 11–19.5 µm (x̄ = 42.3 × 14.5 µm, n = 30), solitary, acrogenous, cylindrical, sometimes clavate, 3–4-septate, constricted and darker at the septa, smooth, brown and rounded at the apex.

##### Culture characteristics.

Conidia germinating on Potato Dextrose Agar (PDA) within 24 h, and producing germ tubes either from the apex or base. Colonies circular, flat, dense, radial sulcate, edge entire, pearl-gray on the surface, dark brown on the reverse and becoming grey-white along the margin.

**Figure 2.**
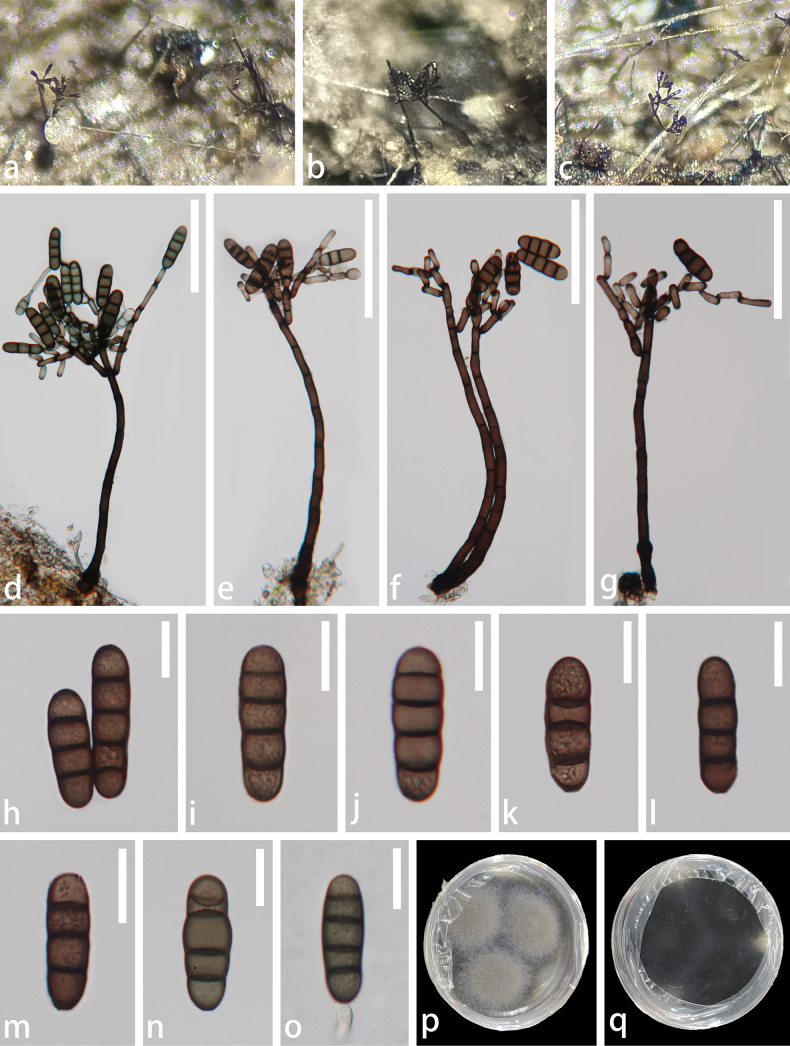
*Kirschsteiniotheliaatra* (GZAAS 23-0807, new host record) **a–c** colonies on natural substrate **d–g** conidiophores and conidiogenous cells bearing conidia **h–n** conidia **o** a germinated conidium **p** upper surface view of culture **q** lower surface view of culture. Scale bars: 100 μm (**d–g**); 20 μm (**h–o**).

##### Material examined.

China • Guizhou Province, Zunyi City, Suiyang County, saprobic on decaying branches of *Edgeworthiachrysantha*, 13 February 2023, Xue-Mei Chen, SY12 (GZAAS 23-0807), living culture GZCC 23-0731.

##### Known distribution (based on molecular data).

China (Su et al. 2016; this study).

##### Known hosts (based on molecular data).

*Edgeworthiachrysantha* (This study), Unidentified decaying wood (Su et al. 2016).

##### Note.

Morphologically, our collection matches the characteristics of *Kirschsteiniotheliaatra*, including macronematous, mononematous conidiophores with numerous short branches; tretic, doliiform, or lageniform conidiogenous cells that develop new cells from the apical or subapical part of the subtending cells; and cylindrical, occasionally clavate conidia that are 3–4-septate, constricted and darker at the septa, which are rounded at the apex (Su et al. 2016). In the phylogenetic analyses, our collection (GZCC 23-0731) clusters with *Kirschsteiniotheliaatra* (CBS 109.53, DEN, MFLUCC 15-0424, MFLUCC 16-1104 and S–783) (Fig. 1). Excluding gaps, no difference was observed in the comparison of nucleotides across the ITS (491 bp), LSU (788 bp) and SSU (844 bp) regions between our collection and *Kirschsteiniotheliaatra* (MFLUCC 16-1104). Based on these findings, we identify our isolate as *Kirschsteiniotheliaatra*, following the guidelines established by Jeewon and Hyde (2016) and Maharachchikumbura et al. (2021). This is the first time *Kirschsteiniotheliaatra* has been reported from *Edgeworthiachrysantha*.

#### ﻿ Kirschsteiniothelia bulbosapicalis

Taxon classificationFungiPleosporalesKirschsteiniotheliaceae

X. Tang, K.D. Hyde, Jayaward. & J.C. Kang, sp. nov.

7F6A1323-2181-545A-9A9D-5A8F92D695F1

Fungal Names number: FN 572044

Facesoffungi number: FoF16485

Fig. 3

##### Etymology.

The specific epithet ‘*bulbosapicalis*’ refers to the bulbous area of the conidia at the apex.

##### Holotype.

GZAAS 23-0808.

##### Description.

***Saprobic*** on unidentified decaying wood. ***Sexual morph***: Undetermined. ***Asexual morph*: *Colonies*** on the natural substrate superficial, effuse, gregarious, hairy, black, glistening. ***Mycelium*** semi-immersed, on the substrate, pale brown to dark brown. ***Conidiophores*** (–47)58–128(–199) μm × 7.5–12.5(–16.5) μm (x̄ = 86.7 × 10.6 μm, n = 15), macronematous, mononematous, solitary, straight or slightly flexuous, cylindrical, unbranched, septate, smooth, brown to dark brown, truncate at the apex and wider at the base. ***Conidiogenous cells*** 6–17 μm × 7–10.5 μm (x̄ = 10.6 × 8.6 μm, n = 15), monoblastic, holoblastic, terminal, determinate, proliferating, cylindrical, brown to dark brown. ***Conidia*** 118–236.5 μm × 15–27 μm (x̄ = 174.8 × 21 μm, n = 30), solitary, acrogenous, cylindrical, ovoid to obclavate, rostrate, smooth, straight or slightly curved, 8–13-septate, slightly constricted at the septa, olivaceous to reddish-brown to dark brown, bulbous at the apex and/or third or fourth cell, truncate at the base, with a spherical hyaline mucilaginous sheath.

**Figure 3.**
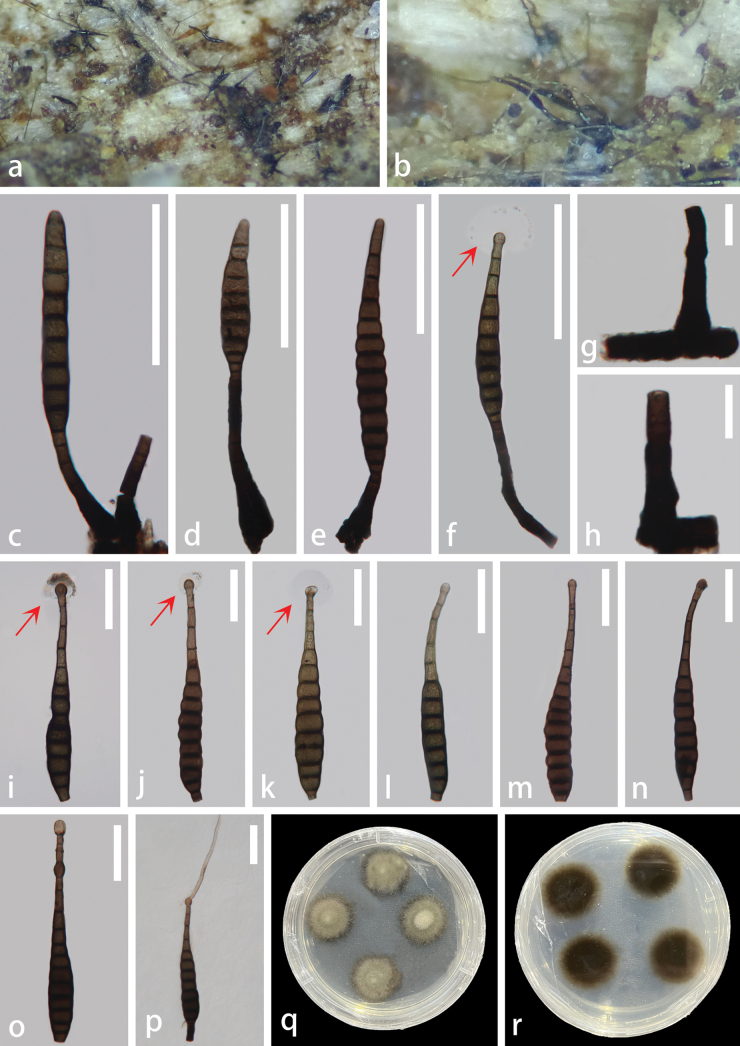
*Kirschsteiniotheliabulbosapicalis* (GZCC 23-0732, holotype) **a, b** colonies natural substrate **c–f** conidiophores, conidiogenous cells bearing conidia (red arrows indicate mucilaginous sheaths) **g, h** conidiophores **i–o** conidia (red arrows indicate mucilaginous sheaths) **p** a germinated conidium **q** upper surface view of culture **r** lower surface view of culture. Scale bars: 100 μm (**c–f**); 20 μm (**g, h**); 50 μm (**i–p**).

##### Culture characteristics.

Conidia germinating on PDA within 24 hours, producing germ tubes from the apex. Colonies displayed a circular morphology with an umbonate elevation, dense growth and a filiform margin. The surface appeared greyish-green, occasionally exhibiting paler mycelium in the bulge region. The reverse colonies exhibited a circular shape with a filiform margin, displaying a dark brown colour, becoming olivaceous towards the periphery.

##### Material examined.

China • Hainan Province, Jianfengling National Forest Park, saprobic on unidentified decaying wood, 23 August 2021, Zili Li, JBT04 (GZAAS 23-0808, holotype), ex-type living culture GZCC 23-0732.

##### Note.

*Kirschsteiniotheliabulbosapicalis* exhibits sporidesmium-like characteristics and shares similar morphologies with other *Kirschsteiniothelia* species. However, *K.bulbosapicalis* can be distinguished from other *Kirschsteiniothelia* species in having different sizes of conidiophores, conidiogenous cells and the unique feature of its conidia, which comprises one or two bulbous structures at or near the apex, with a spherical hyaline mucilaginous sheath. Phylogenetically, *K.bulbosapicalis* is sister to *K.dujuanhuensis* (KUNCC 22-12671) with 85% ML and 0.99 PP support (Fig. 1). Similar to our new species, *K.dujuanhuensis* also comprises a spherical hyaline mucilaginous sheath. *Kirschsteiniotheliabulbosapicalis* is characterised by larger conidiophores [(–47)58.5–128(–199) μm × 7.5–12.5(–16.5) μm, L/W ratio = 8.2] compared to *K.dujuanhuensis* [29–74(–119) × 9–11 μm, L/W ratio = 5.1] and larger conidia (118–236.5 μm × 15–27 μm, L/W ratio = 8.3) compared to *K.dujuanhuensis* [(114–)122–155(–170) × 10–13(–16) μm, L/W ratio = 11.5]. In addition, *K.bulbosapicalis* exhibits cylindrical to ovoid or obclavate conidia with 8–13 septa and often consist of bulbous structures at the apex and/or the third or fourth cell, as well as a spherical hyaline mucilaginous sheath. In contrast, *K.dujuanhuensis* typically contains obclavate to subcylindrical conidia that are 6–15 septate.

In addition, the comparison of the nucleotides between the sequences of *K.bulbosapicalis* and *K.dujuanhuensis* showed differences of 9% (47/512 bp) across ITS, 1% (8/812 bp) across LSU and 0.1% (2/1003 bp) across SSU, excluding gaps. Based on these findings, we introduce *K.bulbosapicalis* as a novel species, in accordance with the guidelines established by Jeewon and Hyde (2016) and Maharachchikumbura et al. (2021).

#### ﻿ Kirschsteiniothelia dendryphioides

Taxon classificationFungiPleosporalesKirschsteiniotheliaceae

X. Tang, K.D. Hyde, Jayaward. & J.C. Kang, sp. nov.

A19BE87A-BA0F-5DD7-BF33-60B1C765E8A2

Fungal Names number: FN 572046

Facesoffungi number: FoF16486

Figs 4 , 5

##### Etymology.

The specific epithet “*dendryphioides*” is derived from the resemblance to the dendryphiopsis-like features.

##### Holotype.

HKAS 136930.

##### Description.

***Saprobic*** on an unidentified decaying wood. ***Sexual morph***: Undetermined. ***Asexual morph*: *Colonies*** on the natural substrate superficial, effuse, scattered, hairy, black, glistening. ***Mycelium*** partly immersed, on the substrate, pale brown to dark brown. ***Conidiophores*** 179–467 × 4.5–8 μm (x̄ = 318.2 × 6.1 μm, n = 10), macronematous, mononematous, erect, subscorpioid branched, straight or flexuous, cylindrical, septate, smooth, brown to dark brown, becoming paler towards the apex. ***Conidiogenous cells*** 9–19 × 4–8 μm (x̄ = 13.3 × 6.1 μm, n = 30), monotretic, terminal or intercalary, integrated, sometimes percurrent, cylindrical, doliiform, mostly discrete, determinate, smooth, pale brown to brown, both ends appearing darker, with new cells developing from the apical or subapical part of the subtending cells. ***Conidia*** 30–55 × 9–13.5 μm (x̄ = 40 × 11.1 µm, n = 30), solitary, acrogenous, cylindrical, oblong and occasionally clavate, smooth, guttulate, 2–4-septate, slightly or deeply constricted and darker at the septa, brown, rounded at the apex and sometimes truncate at the base, exhibiting obtuse ends.

**Figure 4.**
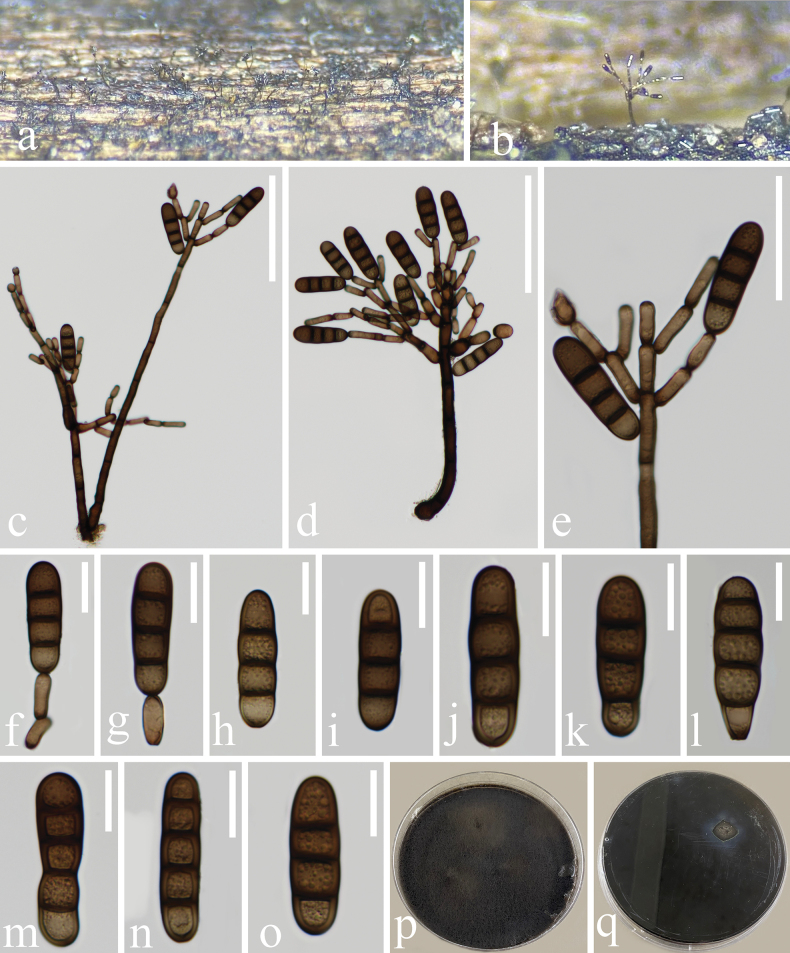
*Kirschsteiniotheliadendryphioides* (HKAS 136930, holotype) **a, b** colonies on natural substrate **c, d** conidiophores, conidiogenous cells bearing conidia **e–g** conidiogenous cells bearing conidia **h–o** conidia **p** upper surface view of culture **q** lower surface view of culture; Scale bars: 100 μm (**c, d**); 50 μm (**e**); 20 μm (**f–o**).

##### Culture characteristics.

Conidia germinating on PDA within 24 hours. Colonies circular, characterised by dense, flat, spreading and fluffy growth, with an entire margin. The surface displayed a dark brown hue, while the reverse colonies exhibited a circular shape with an entire margin, also appearing dark brown.

**Figure 5.**
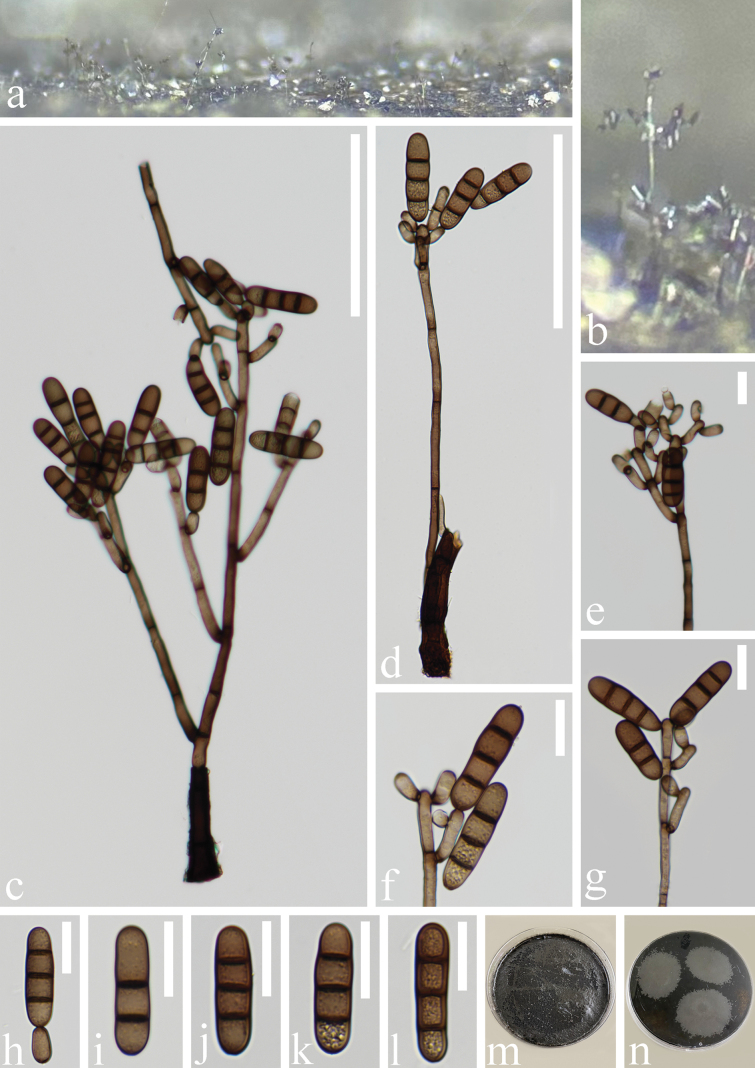
*Kirschsteiniotheliadendryphioides* (HKAS 135651, paratype) **a, b** colonies on natural substrate **c, d** conidiophores, conidiogenous cells bearing conidia **e–g** conidiogenous cells bearing conidia **h–l** conidia **m** upper surface view of culture **n** lower surface view of culture. Scale bars: 100 μm (**c, d**); 20 μm (**e–l**).

##### Material examined.

China • Yunnan Province, Lushui City, Sanhe Village, Gaoligong Mountain, saprobic on decaying wood in a freshwater stream, 5 May 2021, Rong-ju Xu, XS17 (HKAS 136930, holotype), ex-type living culture, KUNCC 10431; *ibid.* • saprobic on submerged decaying wood in freshwater habitats, 22 August 2021, Rong-ju Xu, SYC-05 (HKAS 135651, paratype), living culture, KUNCC 10499.

##### Notes.

*Kirschsteiniotheliadendryphioides* exhibits dendryphiopsis-like characteristics and shares similar morphologies with other *Kirschsteiniothelia* species. However, *K.dendryphioides* differs from other species in the size of its conidiophores, conidiogenous cells and conidia. *Kirschsteiniotheliadendryphioides* is distinct from *K.atra* in having larger conidiophores (179–467 × 4.5–8 μm, L/W ratio = 52.2 vs. 148–228 µm × 6–8 μm, L/W ratio = 27), shorter conidiogenous cells (9–19 × 4–8 μm, L/W ratio = 2.2 vs. 25–33 µm × 5–7 μm, L/W ratio = 4.8) and smaller conidia (30–55 × 9–13.5 μm, L/W ratio = 3.6 vs. 54–63 ×14–18 μm, L/W ratio = 3.4).

The establishment of *Kirschsteiniotheliadendryphioides* as a new species is further supported by molecular data. Based on our phylogenetic analyses, *K.dendryphioides* strains (KUNCC 10431 and KUNCC 10499) form a subclade sister to the strains of *Kirschsteiniotheliaatra* (CBS 109.53, DEN, MFLUCC 15-0424, MFLUCC 16-1104 and S-783) with 83% ML and 0.99 PP support (Fig. 1). The comparison of the nucleotides between the sequences of *K.dendryphioides* and *K.atra* (MFLUCC 16-1104) shows a difference of 1.9% (9/481 bp) across ITS and 2.4% (11/458 bp) across SSU, but no difference was observed across LSU (777 bp), excluding gaps. Based on these findings, we introduce *Kirschsteiniotheliadendryphioides* as a novel species, following guidelines outlined in Jeewon and Hyde (2016) and Maharachchikumbura et al. (2021). We were unable to compare the nucleotide differences across LSU and SSU of KUNCC 10499 as it lacks sequence data for these loci.

#### ﻿ Kirschsteiniothelia longirostrata

Taxon classificationFungiPleosporalesKirschsteiniotheliaceae

X. Tang, K.D. Hyde, Jayaward. & J.C. Kang, sp. nov.

22B93B83-4A45-56CA-9239-434CB037CD93

Fungal Names number: FN 572045

Facesoffungi number: FoF16487

Fig. 6

##### Etymology.

The specific epithet ‘*longirostrata*’ refers to the conidia containing a long rostrate.

##### Holotype.

GZAAS 23-0809.

##### Description.

***Saprobic*** on an unidentified submerged decaying wood. ***Sexual morph***: Undetermined. ***Asexual morph*: *Colonies*** on the natural substrate superficial, effuse, gregarious, hairy, black, glistening. ***Mycelium*** partly immersed on the substrate, composed of branched, septate, smooth-walled hyphae, pale to dark brown. ***Conidiophores*** 80–252 × 4.5–9.5 μm (x̄ = 161.3 × 6.8 μm, n = 20), macronematous, mononematous, solitary, cylindrical, straight, or slightly flexuous, unbranched, percurrent, smooth, guttulate, 4–13-septate, sometimes slightly constricted at the septa, brown to dark brown tapering towards the apex and wider at the base. ***Conidiogenous cells*** 6.5–16 × 5–9 μm (x̄ = 13× 7 μm, n = 20), monoblastic, terminal or indeterminate, percurrently proliferating, cylindrical, pale brown to brown. ***Conidia*** 36.5–109(–160) × 8–16 μm (x̄ = 71× 12 μm, n = 30), solitary, acrogenous, cylindrical, obpyriform to obclavate, rostrate 15–100(–120) × 2.5–6 μm (x̄ = 48 × 4.3 μm, n = 30), smooth, straight or curved, guttulate, 6–18-septate, slightly constricted and darker at the septa, proliferating, pale brown to brown, becoming paler towards the apex, with a truncate base and a mucilaginous sheath surrounding the upper part of the apex.

**Figure 6.**
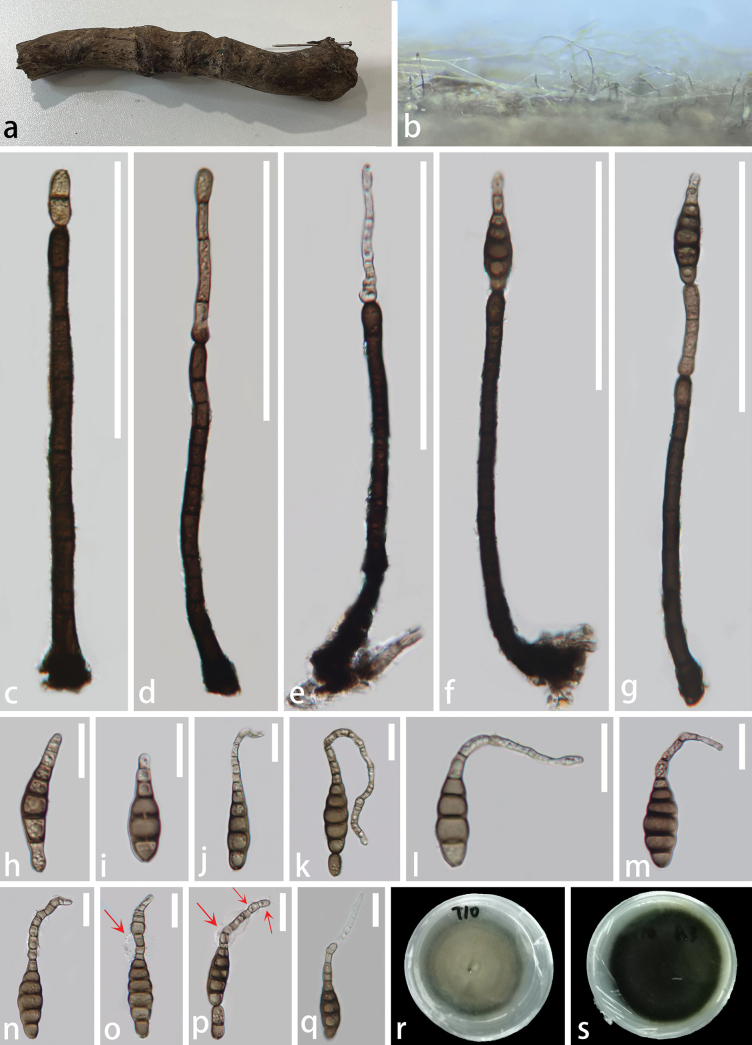
*Kirschsteiniothelialongirosrata* (GZCC 23-0733, holotype) **a** unidentified submerged wood **b** colonies on natural substrate **c, d** conidiophores, conidiogenous cells **e–g** conidiophores, conidiogenous cells bearing conidia **h–p** conidia (red arrows indicate mucilaginous sheaths) **q** a germinated conidium; **r** Upper surface view of culture **s** lower surface view of culture. Scale bars: 100 μm (**c–g**); 20 μm (**h–q**).

##### Culture characteristics.

Conidia germinating on PDA within 24 hours, producing germ tubes from the apex. Colonies displayed a circular morphology with dense, flat, spreading and fluffy growth, with an entire margin. The surface exhibited an olivaceous-green hue with a darker edge, while the reverse colonies displayed a circular shape with an entire margin, appearing blackish-green.

##### Material examined.

China • Hainan Province, Jianfengling National Forest Park, saprobic on submerged unidentified decaying wood, 23 August 2021, Zili Li, T10 (GZAAS 23-0809, holotype) ex-type living culture GZCC 23-0733.

##### Notes.

*Kirschsteiniothelialongirostrata* exhibits sporidesmium-like characteristics and shares similar features with other *Kirschsteiniothelia* species. *Kirschsteiniothelialongirostrata* can be distinguished from other *Kirschsteiniothelia* species in having different sizes and shapes of conidiophores, conidiogenous cells and unique features of conidia, such as obpyriform to obclavate, long rostrate, proliferating, with a mucilaginous sheath surrounding the upper part of the apex. Unlike *K.crustacea*, *K.longirostrata* has cylindrical, proliferating conidiogenous cells and obpyriform to obclavate conidia, with longer (15–100(–120) × 2.5–6 μm), guttulate, proliferating rostrate structures and a mucilaginous sheath surrounding the upper part of the apex.

Molecular data further supports the establishment of *Kirschsteiniothelialongirostrata* as a novel taxon. Based on our phylogenetic analyses, *K.longirostrata* is sister to *K.crustacea* (MFLU 21-0129) with 94% ML and 1.00 PP support (Fig. 1). The comparison of the nucleotides between the sequences of *K.longirostrata* (GZCC 23-0733) and *K.crustacea* (MFLU 21-0129) shows differences of 8.4% (39/467 bp) across ITS and 0.7% (5/718 bp) across LSU, excluding gaps. However, we were unable to compare the nucleotide differences across SSU as *K.crustacea* lacks sequence data for this locus. Based on these findings, we introduce *Kirschsteiniothelialongirostrata* as a novel species, following guidelines outlined in Jeewon and Hyde (2016) and Maharachchikumbura et al. (2021).

## ﻿Discussion

During surveys on saprobic fungi associated with woody plants in the subtropical and tropical forests of the Guizhou, Hainan and Yunnan Provinces in China, we discovered three previously undocumented taxa and one known species, which belong to *Kirschsteiniothelia*. They were found on decaying wood, including some unidentified hosts and *Edgeworthiachrysantha*. All these species have been reported with their asexual morph, either exhibiting the dendryphiopsis-like or sporidesmium-like morphologies.

Our newly-described taxa include two sporidesmium-like species, namely *Kirschsteiniotheliabulbosapicalis* and *K.longirostrata* and one dendryphiopsis-like taxon, *K.dendryphioides*. Both *Kirschsteiniotheliabulbosapicalis* and *K.longirostrata* exhibit distinct characteristics from other species of *Kirschsteiniothelia*. *Kirschsteiniotheliabulbosapicalis* is characterised by acrogenous, cylindrical, ovoid, obclavate, rostrate, straight or slightly curved conidia with 8–13 septa, often bulbous at the apex and/or third or fourth cell, with a spherical hyaline mucilaginous sheath. *Kirschsteiniothelialongirostrata* displays solitary, acrogenous, cylindrical, obpyriform to obclavate, rostrate, smooth, straight or curved and guttulate conidia that are paler towards the apex, consisting of 6–18 septa, slightly constricted and darker at the septa, with a mucilaginous sheath surrounding the tail-like upper part of the apex. *Kirschsteiniothelialongirostrata* has the longest tail amongst all current *Kirschsteiniothelia* species, which proliferates from the apex of the conidium. Our phylogenetic analyses reveal that our new species belong to *Kirschsteiniothelia* with stable support values and, in particular, is closely related to *K.crustacea*.

*Kirschsteiniotheliadendryphioides* displays solitary, acrogenous, cylindrical, oblong and occasionally clavate, smooth, guttulate, 2–4-septate conidia with slightly or deeply constricted and darker at the septa, rounded at the apex and sometimes truncate at the base, exhibiting obtuse ends. However, *Kirschsteiniotheliadendryphioides* is characterised by larger conidiophores, shorter conidiogenous cells and smaller conidia when compared to *K.atra*. Although they are morphologically similar, there are also sufficient dissimilarities in the DNA sequence data.

The new host record for *Kirschsteiniotheliaatra* shows characteristics of solitary, acrogenous, cylindrical, sometimes clavate conidia that are 3–4-septate, constricted and darker at the septa and smooth and rounded at the apex. Based on a comparison of morphological and phylogenetic analyses, no significant differences were observed in the DNA base pairs and the morphological variations fall within the range of intraspecific diversity. Therefore, we identified our new collection (GZCC 23–0731) as the known species *K.atra*. Furthermore, this report extends the known host range of *K.atra* (Table 2).

**Table 2.** *Kirschsteiniothelia* species and data related to their host, habitat, country and reported morph.

Taxa	Host	Habitat	Morphological character	Asexual Morph character	Country	Molecular data	References
*Kirschsteiniotheliaabietina*	*Tsugacanadian*	Terrestrial	Sexual	N/A	USA	N/A	Fairman (1905); Wang et al. (2004)
*K.acerina*	On absorbing mycorrhizal rootlets of *Acersaccharum*	Terrestrial	Sexual	N/A	USA	N/A	Hawksworth (1985)
*K.acutispora*	Unidentified decaying wood	Terrestrial	Asexual	Sporidesmium-like	Thailand	A	Jayawardena et al. (2022)
*K.agumbensis*	On decaying wood of *Garcinia* sp.	Terrestrial	Asexual	Sporidesmium-like	India	A	Sruthi et al. (2024)
*K.atra*	*Abiesbalsamea*, *Acernegundo*, *Acer* sp., *Agathisaustralis*, *Alnusglutinosa*, *A.incana*, *Alstonia* sp., *Betulapapyrifera*, *Brachyglottisrepanda*, *Bursera* sp., *Carpinusbetulus*, *Carpinus* sp., *Celtis* sp., *Clematis* sp., *Coprosmaaustralis*, *Corylusavellana*, *Cupressusmacrocarpa*, *Cupressus* sp., *Drypetesalba*, *Edgeworthiachrysantha*, *Fraxinuspennsylvanica*, *Fraxinus* sp., *Fuchsiaexcorticata*, *Hederahelix*, *Juglans* sp., *Knightiaexcelsa*, *Leptospermumscoparium*, *Loniceracoerulea*, *Machaerocereus* sp., *Macropiperexcelsum*, *Nothofagustruncata*, *Phoenixdactylifera*, *Pinusbanksiana*, *Populusangustifolia*, *P.balsamifera*, *P.tremuloides*, *Prunus* sp., *Quercusrobur*, *Quercus* sp., *Rhopalostylis* sp., *Salix* sp., *Tiliaamericana*, *Tsugacanadensis*, Unidentified decaying wood	Freshwater, terrestrial	Sexual and Asexual	Dendryphiopsis-like	Australia, Belgium, China, Czech Republic, France, Germany, Mexico, New Zealand, Poland, Russian Federation, Sweden, Unite Kingdom, USA	A	Aptroot (1995, 1997); Cannon et al. (1985); Chlebicki and Chmiel (2006); Conners (1967); Cooke (1985); Eriksson (1992, 2014); Ginns (1986); Hughes (1978); Hyde (1993); Kobayashi (2007); Matsushima (1971, 1975); McKenzie et al. (2000, 2004); Minter et al. (2001); Mulenko et al. (2008); Nattrass (1961); Nordén et al. (1997); Popov et al. (2008); Rao and Varghese (1981); Réblová and Svrcek (1997); Schmid-Heckel (1988); Sieber et al. (1995); Sierra (1984); Su et al. (2016); Sutton (1973); This study; Wang and He (2007); Wang (2010); Wang et al. (2004).
*K.aquatica*	Unidentified decaying wood	Freshwater	Asexual	Sporidesmium-like	China	A	Bao et al. (2018)
*K.arasbaranica*	Dead branches of *Quercuspetraea*	Terrestrial	Sexual	N/A	Iran	A	Mehrabi et al. (2017)
*K.arbuscula*	On bark of *Acer*, *Rhuscopallinum*, *Carya*, *Magnoliaglauca*, and *Acerrubrum*	Terrestrial	Asexual	Dendryphiopsis-like	USA	N/A	Berkeley (1875); Ellis (1976); Pratibha et al. (2010); Sruthi et al. (2024)
*K.atkinsonii*	*Freycinetiaarnotti*	Terrestrial	Sexual	N/A	Greece	N/A	Stevens (1925)
*K.binsarensis*	On dead twig	Terrestrial	Asexual	Dendryphiopsis-like	India	N/A	Subramanian and Srivastava (1994); Sruthi et al. (2024)
*K.biseptata*	On dead wood	Terrestrial	Asexual	Dendryphiopsis-like	South Africa	N/A	Morgan-Jones et al. (1983); Sruthi et al. (2024)
*K.bulbosapicalis*	Unidentified decaying wood	Terrestrial	Asexual	Sporidesmium-like	China	A	This study
*K.cangshanensis*	Unidentified decaying wood	Freshwater	Asexual	Sporidesmium-like	China	A	Bao et al. (2018)
*K.chiangmaiensis*	Unidentified decaying wood	Terrestrial	Sexual	N/A	Thailand	A	Louangphan et al. (2024)
*K.crustacea*	Bamboo	Terrestrial	Asexual	Sporidesmium-like	Thailand	A	Jayawardena et al. (2022)
*K.dendryphioides*	Unidentified decaying wood	Freshwater	Asexual	Dendryphiopsis-like	China	A	This study
*K.dolioloides*	*Ramulisdecorticatispineis*	Terrestrial	Sexual	N/A	Switzerland	N/A	Wegelin (1894); Wang et al. (2004)
*K.dujuanhuensis*	Unidentified submerged wood	Freshwater	Asexual	Sporidesmium-like	China	A	Unpublished
*K.dushanensis*	Unidentified decaying wood	Freshwater	Asexual	Sporidesmium-like	China	A	Yang et al. (2023)
*K.ebriosa*	Sparkling wine	N/A	Asexual	Dendryphiopsis-like	Spain	A	Rodríguez-Andrade et al. (2020)
*K.emarceis*	Unidentified decaying wood	Terrestrial	Sexual and asexual	Dendryphiopsis-like	Thailand	A	Boonmee et al. (2012)
*K.esperanzae*	Unidentified decaying wood	Terrestrial	Sexual	N/A	Mexico	N/A	Raymundo et al. (2023)
*K.extensum*	Unidentified decaying wood	Terrestrial	Asexual	Sporidesmium-like	Thailand	A	Jayawardena et al. (2022)
*K.fascicularis*	On bark of *Liquidambar* sp.	Terrestrial	Asexual	Dendryphiopsis-like	USA	N/A	Berkeley (1875); Hughes (1958); Sruthi et al. (2024)
*K.fluminicola*	Unidentified decaying wood	Freshwater	Asexual	Sporidesmium-like	China	A	Bao et al. (2018)
*K.goaensis*	On dead and decaying bark of tree	Terrestrial	Asexual	Dendryphiopsis-like	India	N/A	Pratibha et al. (2010); Sruthi et al. (2024)
*K.guangdongensis*	Dead branches of unidentified plant	Terrestrial	Asexual	Sporidesmium-like	China	A	Senanayake et al. (2023)
*K.inthanonensis*	Unidentified decaying wood	Terrestrial	Asexual	Dendryphiopsis-like	Thailand	A	de Farias et al. (2024)
*K.laojunensis*	Bark of *Abiesfabri*	Terrestrial	sexual	N/A	China	A	Meng et al. (2024)
*K.lignicola*	Unidentified decaying wood	Terrestrial	Sexual and Asexual	Dendryphiopsis-like	Thailand	A	Boonmee et al. (2012)
*K.longirostrata*	Unidentified submerged decaying wood	Freshwater	Asexual	Sporidesmium-like	China	A	This study
*K.longisporum*	Dead branches of *Pinustaeda*	Terrestrial	Asexual	Dendryphiopsis-like	China	A	Tian et al. (2024)
*K.nabanheensis*	Unidentified broadleaf tree	Terrestrial	Asexual	Dendryphiopsis-like	China	A	Liu et al. (2023b)
*K.phileura*	*Tiliaamericana*	Terrestrial	Sexual	N/A	USA	N/A	Cooke and Ellis (1876); Saccardo (1882)
*K.phoenicis*	*Phoenixpaludosa*	Marine	Sexual	N/A	Thailand	A	Hyde et al. (2018)
*K.pini*	On decaying branches of a *Pinus* sp.	Terrestrial	Asexual	Sporidesmium-like	China	A	Jin et al. (2024)
*K.populi*	*Populusangustifolia*	Terrestrial	Sexual	N/A	USA	N/A	Greene (1901); Wang et al. (2004)
*K.proteae*	*Proteacynaroides*	N/A	Sexual	N/A	South Africa	N/A	Marincowitz et al. (2008)
*K.puerensis*	Coffee wood	Terrestrial	Asexual	Sporidesmium-like	China	A	Hyde et al. (2023)
*K.ramus*	Unidentified decaying wood	Freshwater	Asexual	Dendryphiopsis-like	China	A	Zhang et al. (2023)
*K.recessa*	Unidentified decaying wood, *Acerrubrum*, *Alnusrubra*, *Pyrus* sp., rotten wood	Terrestrial	Sexual and asexual	Dendryphiopsis-like	Canada, Italy, USA,	N/A	Hawksworth (1985); Barr et al. (1986); Aptroot (1995); Wang et al. (2004)
*K.reticulata*	Unidentified twigs	Terrestrial	Sexual	N/A	China	N/A	Chen et al. (2006)
*K.rostrata*	Unidentified decaying wood	Freshwater	Asexual	Sporidesmium-like	China	A	Bao et al. (2018)
*K.saprophytica*	Unidentified decaying wood	Terrestrial	Sexual and asexual	Dendryphiopsis-like	Thailand	A	de Farias et al. (2024)
*K.septemseptatum*	Unidentified decaying wood	Terrestrial	Asexual	Dendryphiopsis-like	Thailand	A	Jayawardena et al. (2022)
*K.sichuanensis*	On decaying branches of an unidentified woody plant	Terrestrial	Asexual	Sporidesmium-like	China	A	Jin et al. (2024)
*K.shimlaensis*	*Cedrusdeodara*	Terrestrial	Asexual	Dendryphiopsis-like	India	N/A	Verma et al. (2021)
*K.smilacis*	*Smilax* sp.	Terrestrial	Sexual	N/A	China	N/A	Chen et al. (2006)
*K.spatiosum*	Unidentified decaying wood	Terrestrial	Asexual	Sporidesmium-like	Thailand	A	Jayawardena et al. (2022)
*K.striatispora*	*Juniperuscommunis*	Terrestrial	Sexual	N/A	China, Switzerland	N/A	Hawksworth (1985)
*K.submersa*	Unidentified decaying wood	Freshwater	Asexual	Sporidesmium-like	China	A	Su et al. (2016)
*K.tectonae*	*Microcospaniculata*, *Tectonagrandis*	Terrestrial	Asexual	Sporidesmium-like	Thailand	A	Li et al. (2016); de Farias et al. (2024)
*K.thailandica*	*Ficusmicrocarpa*	Terrestrial	Asexual	Sporidesmium-like	Thailand	A	Sun et al. (2021)
*K.thujina*	*Abiesbalsamea*, *Thujaoccidentalis*	Terrestrial	Sexual	N/A	Canada, USA	A	Saccardo (1882); Hawksworth (1985)
*K.umbrinoidea*	*Aesculushippocastanum*	Terrestrial	Sexual	N/A	Italy	N/A	Passerini (1887); Wang et al. (2004)
*K.vinigena*	Cork stopper, sparkling wine	N/A	Asexual	Dendryphiopsis-like	Spain	A	Rodríguez-Andrade et al. (2020)
*K.xera*	*Prunus* sp.	Terrestrial	Sexual	N/A	USA	N/A	Fairman (1910); Wang et al. (2004)
*K.xishuangbannaensis*	*Heveabrasiliensis*	Terrestrial	Asexual	Sporidesmium-like	China	A	Xu et al. (2023)
*K.zizyphifolii*	*Nayariophytonzizyphifolium*	Terrestrial	Sexual and asexual	Dendryphiopsis-like	Thailand	A	de Farias et al. (2024)

**N/A**: data not available; **A**: data available.

Whether we can use sporidesmium-like and dendryphiopsis-like morphs to differentiate species is currently obscure. Based on our phylogeny, species with sporidesmium-like and dendryphiopsis-like morphs do constitute distinct clades. There are three dendryphiopsis-like species (*K.inthanonensis*, *K.nabanheensis* and *K.septemseptatum*) that constitute a strongly-supported subclade, but they are nested within sporidesmium-like species. Therefore, segregating species based on this aspect should be dealt with caution. As with other asexual fungi, some species of *Kirschsteiniothelia* occur solely in the sexual morph and, hence, we are unable to compare their morphologies with other asexual species.

Besides establishing three novel *Kirschsteiniothelia* species and a new host record, we provide a checklist of all *Kirschsteiniothelia* taxa (Table 2), which incorporate 41 asexual morph species, amongst which 21 exhibit the sporidesmium-like morph, while 20 display the dendryphiopsis-like features (Hawksworth 1985; Boonmee et al. 2012; Su et al. 2016; Sun et al. 2021; Xu et al. 2023; de Farias et al. 2024; Jin et al. 2024; Tian et al. 2024). This checklist includes data on the host, habitat preferences, reported morphology, country of origin and availability of molecular data for all species of *Kirschsteiniothelia*. The checklist also provides the latest ecological information of species in the genus.

From the checklist (Table 2), we decipher that most *Kirschsteiniothelia* species have been reported from China (23 species), followed by Thailand (14 species), with a few distributed across different countries including Australia, Belgium, Canada, Czechia, France, Germany, Greece, Italy, India, Iran, Mexico, New Zealand, Poland, Russian Federation, Sweden, Switzerland, South Africa, Spain, United Kingdom and USA (Boonmee et al. 2012; Mehrabi et al. 2017; Bao et al. 2018; Rodríguez-Andrade et al. 2020; Jayawardena et al. 2022; Senanayake et al. 2023; Liu et al. 2023b; Yang et al. 2023; de Farias et al. 2024; Louangphan et al. 2024). As noted in Table 2, the proportion of new species of *Kirschsteiniothelia* discovery in China and Thailand reaches 63%, while other countries and regions have only sporadically discovered one or two species. Hence, we presume that *Kirschsteiniothelia* is highly diverse with many potentially more unknown species and other tropical and subtropical regions that should be explored. In addition, most new species in China and Thailand were primarily found in tropical and subtropical regions, with nearly all species growing on decayed wood (Bao et al. 2018; Rodríguez-Andrade et al. 2020; Sun et al. 2021; Jayawardena et al. 2022; Senanayake et al. 2023; Liu et al. 2023b; Yang et al. 2023; de Farias et al. 2024; Louangphan et al. 2024). We speculate that the high proportion of new species discovered in China and Thailand can be attributed to the following reasons: 1) The tropical/subtropical climatic conditions in China and Thailand are suitable for the growth of these species, with optimal temperature and humidity levels that favour spore germination and colony growth; 2) Samples of *Kirschsteiniothelia* are easily observed on natural substrates and easy to collect; 3) They proliferate quite easily and are potentially good decomposers of substrates and they grow well on most common culture media without requiring specific cultivation conditions; 4) There are many mycologists in China and Thailand who are actively involved in fungal taxonomy and are more likely to discover more new species.

Most species of *Kirschsteiniothelia* are saprobes occurring mainly in terrestrial and followed by freshwater habitats, with only a few taxa reported from environments, such as cork stoppers and as a pathogen that infects human beings (Hawksworth 1985; Boonmee et al. 2012; Su et al. 2016; Nishi et al. 2018; Rodríguez-Andrade et al. 2020; Sun et al. 2021; Xu et al. 2023; de Farias et al. 2024). According to our results, species of *Kirschsteiniothelia* are also present in various habitats, albeit in small numbers. That may be because most studies have overlooked these more specific habitats. We recommend that future research should focus on exploring this genus in diverse environments to potentially discover additional species. In addition, most of the newly-discovered species are asexual on decayed wood samples. The latter provides ample organic nutrients which favour the emergence of the asexual morph and allows them to colonise new areas and propagate (Zalamea et al. 2016; Liu et al. 2023a).

From a morphological perspective, the sporidesmium-like species are more diverse compared to the dendryphiopsis-like taxa. Interspecies differences are mainly attributed to the number and size of the conidiophores, conidiogenous cells and septa in the conidia. However, relying only on these features for species delineation is challenging and insufficient. Prior to the incorporation of molecular data, the taxonomy of *Kirschsteiniothelia* was challenging, resulting in controversial classifications. This genus was initially accommodated in Pleosporaceae by Hawksworth (1985) and Barr (1987), but later transferred to Pleomassariaceae by Barr (1993), based on morphological data. Based on molecular data, Schoch et al. (2006) suggested that *Kirschsteiniothelia* does not belong to any family within Pleosporales, but would rather be in a new family. Subsequently, Boonmee et al. (2012) introduced Kirschsteiniotheliaceae to accommodate *Kirschsteiniothelia* species, based on morphology and phylogenetic analyses. Hernández-Restrepo et al. (2017) established a novel order, Kirschsteiniotheliales, to accommodate the Kirschsteiniotheliaceae taxa. Subsequent studies have followed this classification, using ITS, LSU and SSU rDNA sequence data in their phylogenies (Sun et al. 2021; Xu et al. 2023; de Farias et al. 2024).

At present, most studies use LSU, ITS and SSU rDNA genes for inferring phylogeny relationships amongst *Kirschsteiniothelia* species. Despite close morphological similarities and overlap amongst species, we noted that there are rather unexpected sequence dissimilarities across the different genes analysed here. We presume that, despite high morphological similarities, these asexual species are characterised by high genetic diversity. This difference in genetic trait presumably enables them to adapt and flourish in different environments, gives them better chances of survival and drives speciation. So far, the ITS, LSU and SSU rDNA genes have most commonly been used to identify species within this genus, but as the number of species increases, we recommend incorporating protein genes like *tub*, *tef1-α* and *rpb2* (Badotti et al. 2017).

## Supplementary Material

XML Treatment for Kirschsteiniothelia atra

XML Treatment for Kirschsteiniothelia bulbosapicalis

XML Treatment for Kirschsteiniothelia dendryphioides

XML Treatment for Kirschsteiniothelia longirostrata

## ﻿Additional information

### Author ORCIDs

Xia Tang https://orcid.org/0000-0003-2705-604X

Rajesh Jeewon https://orcid.org/0000-0002-8563-957X

Ruvishika S. Jayawardena https://orcid.org/0000-0001-7702-4885

Deecksha Gomdola https://orcid.org/0000-0002-0817-1555

Yong-Zhong Lu https://orcid.org/0000-0002-1033-5782

Rong-Ju Xu https://orcid.org/0000-0002-3968-8442

Abdulwahed Fahad Alrefaei https://orcid.org/0000-0002-3761-6656

Fatimah Alotibi https://orcid.org/0000-0003-3629-5755

Kevin D. Hyde https://orcid.org/0000-0002-2191-0762

Ji-Chuan Kang https://orcid.org/0000-0002-6294-5793

### Data availability

All the data that support the findings of this study are available in the main text. DNA sequences generated have been submitted to GenBank.
